# A novel homoarginine-containing cyclic peptide pioamide with selective antipseudomonal activity isolated from the nematode symbiont *Photorhabdus khanii*

**DOI:** 10.1128/aem.01123-25

**Published:** 2025-09-24

**Authors:** Yu Imai, Sangkeun Son, Miho Sasaki, Libang Liang, Michael F. Gates, Meghan Ghiglieri, Takeshi Shimosato, Chandrashekhar Honrao, Xiaoyu Ma, Jason J. Guo, Kim Lewis

**Affiliations:** 1 Institute for Aqua Regeneration, Shinshu University, Nagano, Japan; 2 Department of Biology, Antimicrobial Discovery Center, Northeastern University 1848, Boston, Massachusetts, USA; 3 Department of Pharmaceutical Sciences, Center for Drug Discovery, Northeastern University 1848, Boston, Massachusetts, USA; 4 Department of Chemistry and Chemical Biology, Barnett Institute for Chemical and Biological Analysis, Northeastern University 1848, Boston, Massachusetts, USA; Universidad de los Andes, Bogotá, Colombia

**Keywords:** *Pseudomonas aeruginosa*, nematode symbionts, *Photorhabdus*, antibiotic

## Abstract

Gram-negative bacteria harbor an outer membrane that physically protects them from the penetration of antibiotics into the cells. This barrier makes it challenging to develop antibiotics that effectively kill Gram-negative pathogens. The entomopathogenic bacterium *Photorhabdus* species produces various bioactive molecules and is receiving attention as an attractive source of novel antibiotics. We identified a novel antipseudomonal antibiotic, pioamide, from the culture supernatant of *Photorhabdus khanii* HGB1456, a strain that produces darobactin, which selectively kills Gram-negative bacteria. Pioamide, a pentapeptide antibiotic with a molecular weight of 704, exhibits selective activity against *Pseudomonas aeruginosa* but does not exhibit any activity against other bacteria or human cell lines. Whole-genome sequencing of spontaneous pioamide-resistant mutants of *P. aeruginosa* revealed mutations in *pmrB*, which encodes a two-component regulatory system response regulator that modifies the lipopolysaccharide composition in Gram-negative bacteria, conferring pioamide resistance to *P. aeruginosa*. Furthermore, the susceptibility of both *P. aeruginosa* PAO1 and the mutant strain PΔ6-Pore, which overexpresses porins and lacks six efflux pumps, to pioamide was identical, indicating that porins and the efflux pump exert no significant effect on the activity of pioamide. These results suggest that pioamide either targets the cell surface of *P. aeruginosa* or is incorporated via a species-specific uptake mechanism. Our findings highlight the potential of *Photorhabdus* strains as an attractive source for the discovery of antibiotics active against Gram-negative pathogens.

**IMPORTANCE:**

The rise of multidrug-resistant Gram-negative bacteria is a growing threat to global public health. Narrow-spectrum antibiotics minimize disruption of the host microbiota and reduce the risk of resistance development in off-target bacteria. In the field of antibacterial discovery, developing compounds effective against *Pseudomonas aeruginosa* remains particularly challenging. Although *Photorhabdus* species are known to produce various antibiotics, their potential remains largely underexplored. In this study, we applied differential screening to a highly concentrated culture extract of *Photorhabdus khanii* HGB1456 and discovered pioamide, a novel cyclic peptide with unusual selective activity against *P. aeruginosa*. Mutations in pmrB confer pioamide resistance to *P. aeruginosa*. However, the mechanism of action is distinct from that of colistin, which also involves resistance conferred by *pmrB* mutations. These findings underscore the untapped potential of *Photorhabdus* species as an attractive source of species-specific antibiotics and highlight the utility of differential screening for discovering compounds with targeted antibacterial activity.

## INTRODUCTION

We are facing an antibiotic resistance crisis (1). *Pseudomonas aeruginosa*, a Gram-negative opportunistic pathogen, possesses a well-developed multidrug efflux pump system and an outer membrane that serves as a barrier against large molecules. These characteristics make *P. aeruginosa* one of the most challenging pathogens to treat with antibiotics, especially in cases of multidrug-resistant (MDR) *P. aeruginosa* infections. In 2024, the World Health Organization classified *P. aeruginosa* as a “High Priority” pathogen that requires new antibiotics (2, 3). Moreover, according to the CDC’s Antibiotic Resistance Threats in the United States 2019, there were 32,600 cases of infection in hospitalized patients, and 2,700 cases resulted in deaths due to MDR *P. aeruginosa* (4).

The entomopathogenic bacterium *Photorhabdus* species is known to produce various bioactive compounds, including antibiotics (5). However, no *Photorhabdus*-derived antibiotics are currently in practical use. Narrow-spectrum antibiotics are preferable for drug development because they reduce the risk of generating drug-resistant mutants in off-target bacteria and minimize disruption of the gut microbiome, which includes beneficial bacteria, such as *Lactobacillus* and *Bifidobacterium. P. aeruginosa* is primarily associated with respiratory and systemic infections. However, systematically or orally administered antibiotics distribute throughout the body, impacting the gut microbiota. Therefore, the development of antibiotics with selective activity against *P. aeruginosa* is essential to mitigate collateral damage to commensal microbiota. We recently introduced the differential screening for antibiotic discovery, which compares the activity of compounds in bacterial culture supernatants against several pathogens to identify compounds with selective activity against specific pathogens (6–8). By applying differential screening with *Photorhabdus* strains as a screening source, we discovered several antibiotics that exhibit selective activity against Gram-negative pathogens. For instance, darobactins and dynobactins, novel classes of antibiotics discovered from *Photorhabdus khanii* and *Photorhabdus australis*, respectively, selectively kill Gram-negative pathogens by inhibiting BamA, a protein involved in inserting nascent outer membrane proteins into the outer membrane (9–11). 3′-Amino-3′-deoxyguanosine (ADG), which was discovered from *Photorhabdus luminescens*, acts as a prodrug that mimics GTP and selectively kills specific Gram-negative bacteria, such as *Escherichia coli* and *Klebsiella pneumoniae*, by interrupting transcription (12). More recently, we rediscovered 3,6-dihydroxy-1,2-benzisoxazole (DHB) from the culture supernatant of *Photorhabdus laumondii* (13). DHB is a known antibiotic with broad activity against Gram-negative bacteria that binds to 4-hydroxybenzoate octaprenyltransferase and inhibits the ubiquinone biosynthesis pathway. These discoveries emphasize the potential of *Photorhabdus* strains as a source of diverse anti-Gram-negative antibiotics and indicate that the combination of differential screening and *Photorhabdus* strains is a promising strategy for discovering novel antibiotics with pathogen-selective activity. Furthermore, most *Photorhabdus* strains harbor more than 20 biosynthetic gene clusters (BGCs) in their genome, with the majority of these clusters encoding unknown compounds (14). We hypothesized that *Photorhabdus* strains could produce additional antibiotics with selective activity against Gram-negative bacteria beyond darobactins, dynobactins, ADG, and DHB. In this study, we report the discovery of pioamide, a novel cyclic pentapeptide antibiotic with selective activity against *P. aeruginosa*. Pioamide exhibits narrow-spectrum activity and likely acts on the cell surface of *P. aeruginosa* or is taken up through a species-specific uptake mechanism.

## MATERIALS AND METHODS

### Isolation of pioamide

*P. khanii* HGB1456 was cultured in 2-L Erlenmeyer flasks containing 1 L tryptic soy broth at 28°C with shaking at 200 rpm for 10–14 days. After the culture, the bacterial cells were removed by centrifugation at 8,000 × *g* for 10 min, and the resulting culture supernatant was semipurified using XAD16N resin (20–60 mesh, Sigma-Aldrich), followed by cation-exchange chromatography (SP Sepharose XL, GE Healthcare), as described previously (9). The active fraction was eluted with 50 mM ammonium acetate buffer (pH 7.0). A 4-mL sample of the highly concentrated semipurified extract, corresponding to 500 mL to 1 L of the culture supernatant, was subjected to reverse-phase high-performance liquid chromatography (HPLC) using a C18 column (Luna 5 µm C18(2) 100 Å, LC column 250 × 21.2 mm, Phenomenex) under the following HPLC conditions: solvent A, Milli-Q water with 0.1% (vol/vol) formic acid (FA); solvent B, acetonitrile (ACN) with 0.1% (vol/vol) FA. The initial concentration of 2% solvent B was maintained for 7 min, followed by a linear gradient to 40% over 23 min at a flow rate of 9 mL/min. UV detection was performed at 254 nm, and fractions were collected every 20 s. The active fractions were eluted between 17 and 19 min. Active fractions from the first HPLC step were further purified by reverse-phase HPLC using a C18 column (XBridge, BEH C18 OBD column, 100 Å, 5 μm, 250 mm × 4.6 mm, Waters) under the following HPLC conditions: solvent A, 50 mM ammonium acetate (pH 8.0); solvent B, ACN. The initial concentration of 2% solvent B was maintained for 2 min, followed by a linear gradient to 95% solvent B over 20 min at a flow rate of 1 mL/min. UV detection was performed at 254 nm. The active compound was eluted at 8.5 min. The purified active fraction was subjected to a final round of purification by reverse-phase HPLC on a C18 column (XBridge, BEH C18 OBD column, 100 Å, 5 μm, 250 mm × 4.6 mm, Waters) under the following HPLC conditions: solvent A, Milli-Q water with 0.1% (vol/vol) FA; solvent B, ACN with 0.1% (vol/vol) FA. The initial concentration of 2% solvent B was maintained for 2 min, followed by a linear gradient to 95% solvent B over 20 min at a flow rate of 1 mL/min. UV detection was performed at 280 nm. The pure active compound was eluted at 7 min.

The activity of HPLC fractions and pioamide was determined by a diffusion assay on agar plates prepared as follows (15). Exponentially growing cultures of *P. aeruginosa* PAO1, *E. coli* MG1655, and *Staphylococcus aureus* HG003 (OD_600_ 0.1–0.9) were diluted to OD_600_ 0.03 in Mueller Hinton II Broth (MHIIB) and then used to cover Mueller Hinton II agar (MHIIA) plates. Excess culture was removed, the plates were dried on a clean bench, and the HPLC fractions or pioamide were spotted onto the MHIIA plates. The plates were incubated overnight at 37°C, and antibacterial activity was evaluated by observing the zone of inhibition around each spot.

### Elucidation of structure

The 1D ^13^C nuclear magnetic resonance (NMR) spectra were acquired on a Bruker AVANCE II 400 equipped with a 5 mm BBFO probe, and all other NMR data were recorded on a Bruker AVANCE II 700 MHz NMR spectrometer equipped with a 5 mm TXI probe. Pioamide was prepared in 500 µL of DMSO-*d*_6_. The spectra were obtained at 300 K, including ^1^H (zg), ^13^C (zgpg30), COSY (cosygpmfqf), TOCSY (dipsi2etgpsi), ^1^H–^15^N HSQC (hsqcetf3gpsi), ^1^H–^13^C HSQC (hsqcedetgpsisp2.3), ^1^H–^13^C HMBC (hmbcgplpndprqf), and ROESY (roesyphpp.2). High-resolution mass spectrometry analysis was performed using a Thermo Scientific LTQ-Orbitrap mass spectrometer equipped with an electrospray ionization (ESI) source in positive ion mode (FTMS ESI positive mode, full scan MS range: m/z 150–2,000). The compound was introduced via syringe infusion. The spray solvent was a binary mixture of water and acetonitrile (80:20, vol/vol) containing 0.1% FA.

### Minimum inhibitory concentration and cytotoxicity assays

The minimum inhibitory concentration (MIC) and cytotoxicity were determined using microbroth dilution as described previously (9). Overnight cultures of *P. aeruginosa* strains, *E. coli* MG1655, *Acinetobacter baumannii* ATCC17978, *K. pneumoniae* ATCC700603, and *S. aureus* HG003 were diluted 1:100 in MHIIB and incubated at 37°C with aeration at 220 rpm. Regarding the *P. aeruginosa* PΔ6-Pore mutant, the strain was cultured in the presence of 30 µg/mL gentamicin, and the expression of porin was induced by adding 100 µM IPTG in MHIIB. Exponential cultures (OD_600_ 0.1–0.9) were diluted to OD_600_ 0.001 (approximately 5 × 10^5^ CFUs/mL) in MHIIB, and 100 µL aliquots were transferred into round-bottomed 96-well plates containing pioamide, which was serially diluted twofold. After overnight incubation at 37°C, the MIC of pioamide was determined as the minimum concentration at which bacterial growth was significantly inhibited. Cytotoxicity was determined using the microplate Alamar Blue assay (MABA/resazurin). Exponentially growing FaDu pharynx squamous cell carcinoma (ATCC HTB-43) and HepG2 liver hepatocellular carcinoma (ATCC HB-8065) cell lines were cultured in Eagle’s minimum essential medium supplemented with 10% fetal bovine serum and then seeded into a 96-well, flat-bottomed, tissue culture plate (Corning), followed by incubation at 37°C with 5% CO_2_. After 24 h, the medium was aspirated and replaced with fresh medium containing the test compounds (twofold serial dilution in 100 µL of media). After 72 h of incubation at 37°C with 5% CO_2_, resazurin (Acros Organics) was added to each well to a final concentration of 0.15 mM. After 3 h, absorbance values were measured at 544 and 590 nm using a BioTek Synergy H1 microplate reader. All MIC and cytotoxicity assays were performed at least in triplicate.

### Fluorescence microscopy

For time-lapse microscopy, *P. aeruginosa* PAO1 was cultured in MHIIB overnight to the stationary phase, and the following day was inoculated into fresh MHIIB at 1:100 and grown for 2 h at 37°C. Cells were diluted 10-fold in MHIIB, placed on top of a 1.5% low-melting-point agarose MHIIB pad containing pioamide (8× MIC), FM4-64 (10 µg/mL), and SYTOX Green (0.5 µM), and observed with a Nikon Ti2-E fluorescence microscope using a 100× oil-immersion objective. The fluorescence signals for SYTOX Green and FM4-64 were collected sequentially by excitation at 480 and 550 nm, respectively, alongside a phase-contrast image. A thermostatic chamber was used to maintain a temperature of 37°C for the duration of the experiment. Images were acquired with NIS-Elements every 30 min at a resolution of 2,048 by 2,048. The images shown in Fig. 4 were processed using an enhanced contrast process, and the HyperStackReg plugin was used to correct for the x–y drift.

### Mutation analysis * *

*P. aeruginosa* PAO1 cells obtained from an exponential culture were washed with PBS and then inoculated onto MHIIA plates containing an HPLC fraction consisting of four times the MIC of pioamide at a density of 6.6 × 10^8^–7.2 × 10^8^ CFUs/plate. After 2 days of incubation at 37°C, spontaneous pioamide-resistant mutant strains were restreaked onto MHIIA plates containing four times the MIC of pioamide to evaluate the stability of resistance. Experiments were conducted with three independent cultures. Genome sequencing and variant calling were conducted at the Microbial Genome Sequencing Center (MiGS, Pittsburgh). Whole-genome sequencing was performed using paired-end reads (2× 150 bp) on Illumina NextSeq 550, and variant calling was conducted using the *P. aeruginosa* genome reference (NCBI: GCF_000006765.1).

## RESULTS AND DISCUSSION

### Discovery of pioamide from *P. khanii* culture

**Fig 1:**
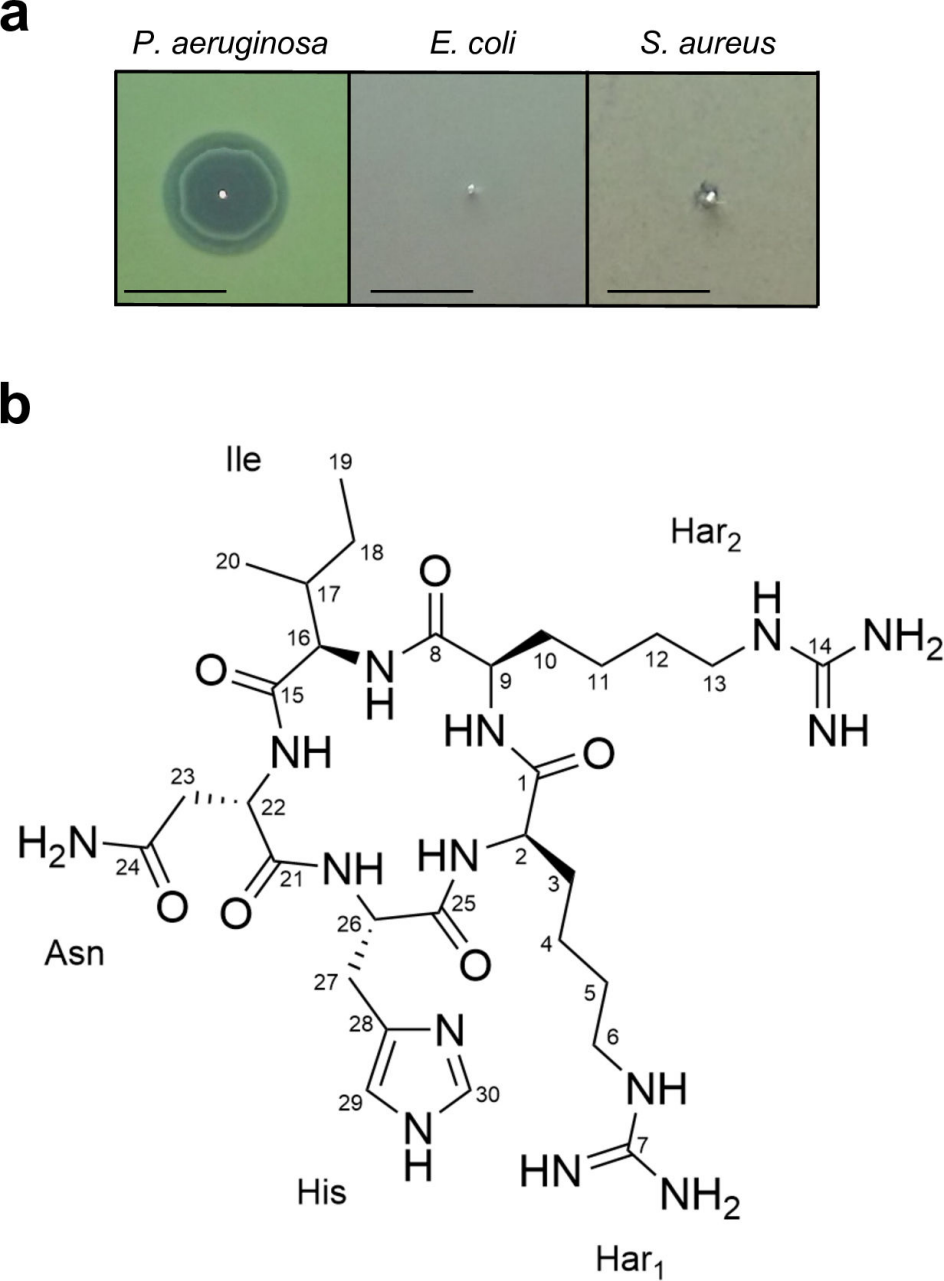
Activity and structure of pioamide. (**a**) Antipseudomonal activity of pioamide. The zone of inhibition indicates its antibacterial activity against *P. aeruginosa*. Scale bar 1 cm. (**b**) Structure of pioamide.

*P. khanii* HGB1456 is a producer of the first member of a new class of antibiotic darobactin, which is encoded by a silent BGC. Genome analysis of *P. khanii* HGB1456 using antiSMASH revealed that this strain harbors 27 BGCs, including the one encoding darobactin (16). Among these, 20 BGCs exhibited low or little similarity to known antibiotics (Table 1). On the basis of this observation, we hypothesized that *P. khanii* HGB1456 has the potential to produce additional antibiotics that selectively kill Gram-negative bacteria, which might be produced in low amounts or encoded by silent BGCs, as in the case of darobactin. To test this hypothesis, we semi-purified the culture supernatant of *P. khanii* HGB1456 using the synthetic resin XAD16N, followed by cation-exchange chromatography. After drying the semi-purified sample, it was resuspended in a small volume of Milli-Q water, resulting in a 250-fold concentration compared with that of the original supernatant, enabling the detection of antibiotic activity from silent BGCs or compounds produced in smaller amounts. This highly concentrated sample was subjected to HPLC, and then the eluents were fractionated. Each fraction was tested for antibacterial activity against the representative Gram-negative bacteria *E. coli* MG1655 and *P. aeruginosa* PAO1, as well as the Gram-positive bacterium *S. aureus* HG003 as a counter screening control. We observed that several fractions demonstrated activity against only *P. aeruginosa* PAO1. Further purification by HPLC combined with bioassay-guided fractionation led to the isolation of a pure active compound that exhibited a distinct zone of inhibition exclusively against *P. aeruginosa* (Fig. 1a; Fig. S1a). The liquid chromatography with tandem mass spectrometry analysis showed a molecular ion at *m*/*z* 705.42 [M+H]^+^ (Fig. S1b). Remarkably, this molecular mass does not match any known compounds listed in the natural product database Antibase. These data suggest that *P. khanii* HGB1456 produces a novel antibiotic.

**TABLE 1:** Biosynthetic gene clusters of secondary metabolites in *P. khanii* HGB1456 predicted by AntiSMASH

Region	Type	Nucleotide start location	Nucleotide end location	Similarity confidence	Most similar known cluster
Region 1.1	NRPS^*a*^	1	46,048		
Region 1.2	NRPS	95,964	223,263	Low	Viscosinamide A/pseudodesmin A (NRPS: Type I)
Region 1.3	NRPS	362,371	472,630		
Region 1.4	NRPS	596,330	640,721		
Region 1.5	NRPS-like, resorcinol	1,143,889	1,215,080	High	Minimycin/indigoidine (NRPS: Type I + saccharide)
Region 1.6	Darobactin	1,231,885	1,253,174	High	Darobactin A (ribosomal: unmodified)
Region 1.7	T1PKS, NRPS	1,355,250	1,404,272		
Region 1.8	NRPS, NRPS-like	1,424,165	1,453,311		
Region 2.1	T1PKS	220,586	265,727		
Region 2.2	Betalactone	340,106	365,596		
Region 2.3	Terpene-precursor	521,928	542,845		
Region 2.4	NRPS	558,658	601,936		
Region 2.5	NRPS, RRE-containing	802,212	879,939	Low	Gamexpeptide A, B, E/luminmide B, D, E, F, G (NRPS: Type I)
Region 3.1	Azole-containing-RiPP	179,457	205,555		
Region 3.2	Phosphonate	340,142	359,150	Low	Luminmycin A/glidobactin A/cepafungin (NRPS: Type I + PKS: Type I)
Region 4.1	PUFA, hglE-KS, NRP-metallophore, NRPS	80,472	192,641	High	Photobactin (NRPS: Type I)
Region 4.2	NRPS	273,698	348,474		
Region 5.1	RiPP-like	7,196	18,119		
Region 5.2	NRPS	105,837	183,376	High	Pyrrolizixenamide A (NRPS: Type I)
Region 5.3	CDPS	259,772	280,479		
Region 7.1	NRPS	173,038	220,182		
Region 8.1	NRPS	73	105,947		
Region 11.1	Leupeptin	32,661	58,419	High	Leupeptin A, B, C, D (other: other)
Region 14.1	Terpene	35,136	60,745	High	Carotenoid (terpene)
Region 15.1	NRPS	15,781	84,883		
Region 19.1	NRPS	16,742	49,564		
Region 23.1	NRPS, NRPS-like	1	18,754	High	Xenematide (NRPS: Type I)

^*a*^ NRPS, nonribosomal peptide synthetase.

### Determination of pioamide structure

**Fig 2:**
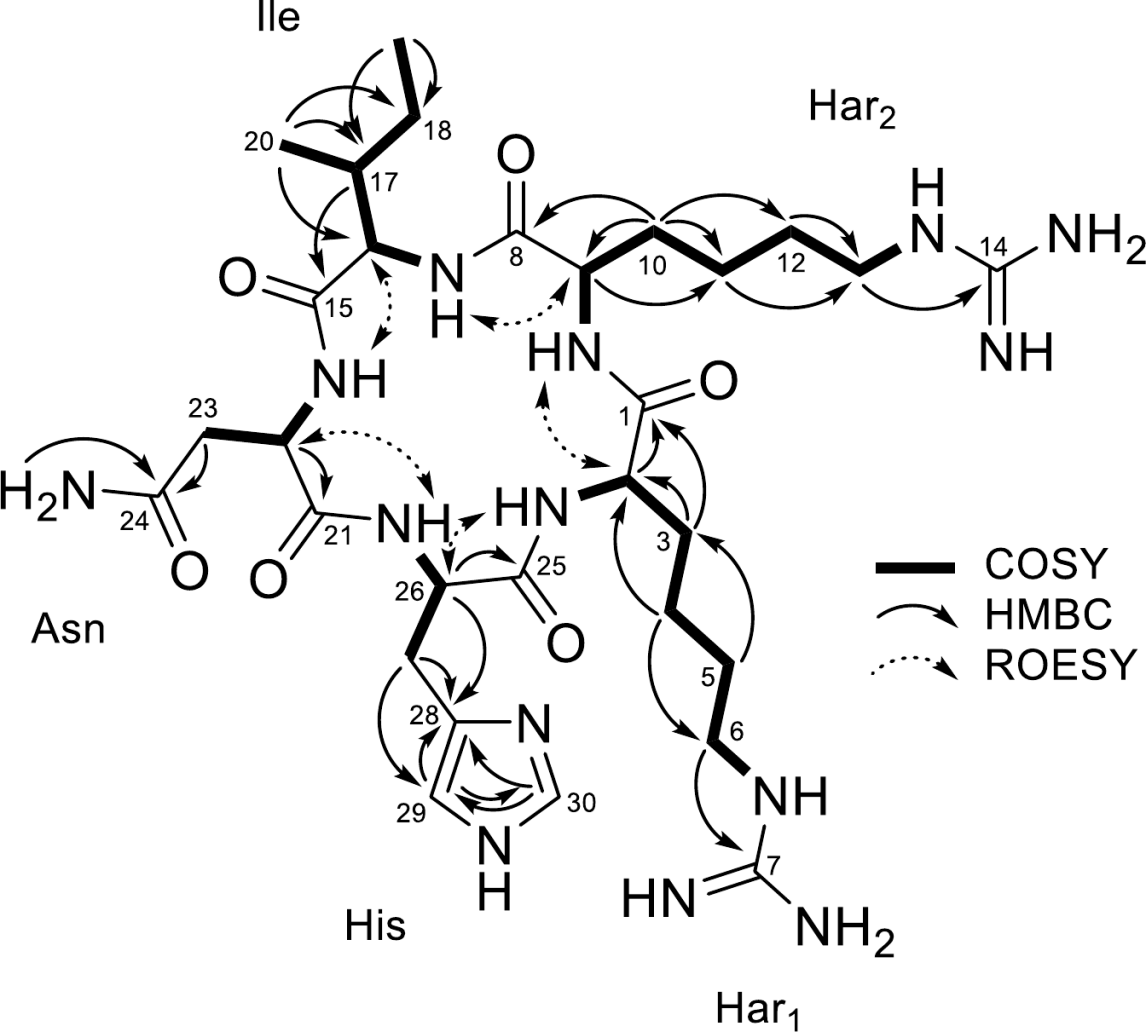
Key 2D NMR correlations of pioamide in DMSO-*d*_6_.

Pioamide was obtained as a white powder. Its structure was determined by MS spectrometric and NMR spectroscopic analysis (Fig. S2). HRESIMS (m/z 705.4208 [M+H]^+^) combined with the NMR data indicated the molecular formula of pioamide as C_30_H_52_N_14_O_6_ (calculated [M+H]^+^ = 705.4267). The analysis of ^1^H, ^13^C, and HSQC NMR spectra indicated the presence of two methyls (δH/C 0.78/14.8 and 0.82/11.4), nine methylenes (δH 1.02–2.83/25.5–31.3 and 2.41/36.7), one aliphatic methine (δH/C 1.72/37.1), and two nitrogen-bearing methylenes (δH/C 3.01/40.4 and 3.03/40.3) (Table S1). The presence of numerous exchangeable protons (δH 6.92–8.71) and downfield chemical shifts of five methine groups (δH/C 3.88/55.1, 4.08/55.7, 4.08/56.5, 4.31/53.1, and 4.36/52.2) strongly indicated the peptidic nature of the compound consisting of five amino acids. A comprehensive analysis of 2D NMR data (COSY, HMBC, and ROESY) determined the structure of each amino acid (Fig. 2). The proton spin system from the amide proton NH-16 (δH 8.02) to the methyl proton H_3_-19 (δH 0.82) through two methines H-16 and H-17, methyl H_3_-20, and methylene H_2_-18 observed in the COSY spectrum and HMBC correlations from H-17 to C-15 revealed the presence of an isoleucine residue. The asparagine residue was determined using the proton spin system (NH-22 to H_2_-23) together with HMBC correlations from H_2_-23 and NH_2_-24 to the amide carbonyl carbon C-24 (δC 170.7). The presence of two closely located carbonyls, C-7 (δC 157.3) and C-14 (δC 157.3), at characteristic chemical shifts indicated the presence of two guanidinium moieties observed in arginine. Further detailed analysis of COSY correlations revealed two spin systems (NH-2/H-2/H_2_-3/H_2_-4/H_2_-5/H_2_-6/NH-6 and NH-9/H-9/H_2_-10/H_2_-11/H_2_-12/H_2_-13/NH-13) consisting of a 4-carbon aliphatic side chain. Furthermore, the clear HMBC correlations from NH-6 and NH-13 to C-7 and C-14 determined the structure of two homoarginine residues, respectively. The remaining proton spin system (NH-26/H-26/H_2_-27) observed in the COSY spectra was extended to the imidazole unit by the key HMBC correlations from H_2_-27 to olefinic carbons C-29 (δC 117.8) and C-28 (δC 132.2). The HMBC correlation of the remaining olefinic proton H-30 (δH 7.49) to the protonated olefinic carbon C-29 and nonprotonated olefinic carbon C-28 clearly established the histidine residue. Finally, analysis of ROESY data revealed five characteristic correlations between α-methines and the amide protons of the preceding residues. Together with the molecular formula, these data demonstrate that pioamide is a cyclic pentapeptide with the sequence: homoarginine-1, homoarginine-2, isoleucine, asparagine, and histidine.

### BGC of pioamide

*P. khanii* HGB1456 harbors 27 distinct BGCs identifiable by the genome accession number WHZZ00000000 using antiSMASH (Table 1). Among these, pioamide, a cyclic pentapeptide compound, is synthesized by a nonribosomal peptide synthetase (NRPS) gene cluster. *Photorhabdus* strains produce antibiotics that are structurally similar to pioamide, collectively referred to as GameXPeptides, which are common secondary metabolites in *Photorhabdus* strains and exhibit extensive activity (17, 18). On the basis of this information, we investigated the BGC responsible for pioamide.

**Fig 3:**
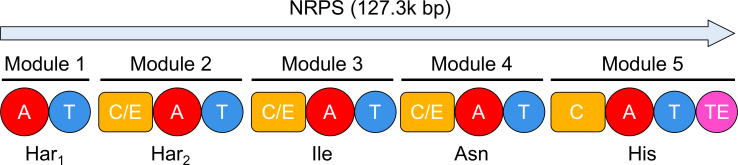
Predicted biosynthetic gene cluster of pioamide. A, adenylation; T, thiolation; C/E, dual condensation/epimerization; C, condensation; TE, thioesterase.

Among the identified BGCs, 17 were NRPS-related clusters. Some of these BGCs feature multiple NRPS genes within a singular cluster, suggesting the potential to encode a variety of nonribosomal peptides. Remarkably, the analysis of adenylation (A) domains revealed two distinct NRPS clusters that encode pentapeptides. The BGC for pioamide was deduced based on both the chemical structure of the compound and the genetic composition of the cluster (Fig. 3). This specific NRPS gene cluster is distinguished by the presence of five A domains containing two adjacent arginine residues. This distinctive A domain configuration strongly implies that the gene cluster region 1.2 is responsible for the production of pioamide (Table 1). The predicted amino acid sequence, Arg-Arg-Ile-X-Tyr, aligned partially with the observed pioamide sequence, identified as Har-Har-Ile-Asn-His (Fig. 1b and 2). The stereochemistry of the α carbons was deduced from the presence of three dual condensation/epimerization (C/E) domains in the modules 2, 3, and 4. The dual C/E domains catalyze the epimerization of the initially bound L-residue into the D-configuration and then promote the condensation of the two residues (17, 19). Thus, the stereochemistry of pioamide was deduced as d-Har, d-Har, d-Ile, l-Asn, and l-His.

### Activity of pioamide

Pioamide exhibits selective activity against *P. aeruginosa* PAO1 with a relatively high MIC of 128 µg/mL (Table S2). Although several antibacterials with activity against *P. aeruginosa* have been reported, only the synthetic compound murepavadin exhibits selective activity against this pathogen (Table S3). Moreover, pioamide exhibited no activity against other bacterial species or human cell lines. Interestingly, we found that the MIC of pioamide against the wild-type strain PAO1 was similar to that against the PΔ6-pore mutant strain, which lacks six efflux pumps (*mex*AB-*opr*M, *mex*CD-*opr*J, *mex*XY, *mex*JKL, *mex*EF-*opr*N, and *tri*ABC) and produces porins in its outer membrane, making it highly susceptible to multiple antibiotics (Table S2) (20, 21).

**Fig 4:**
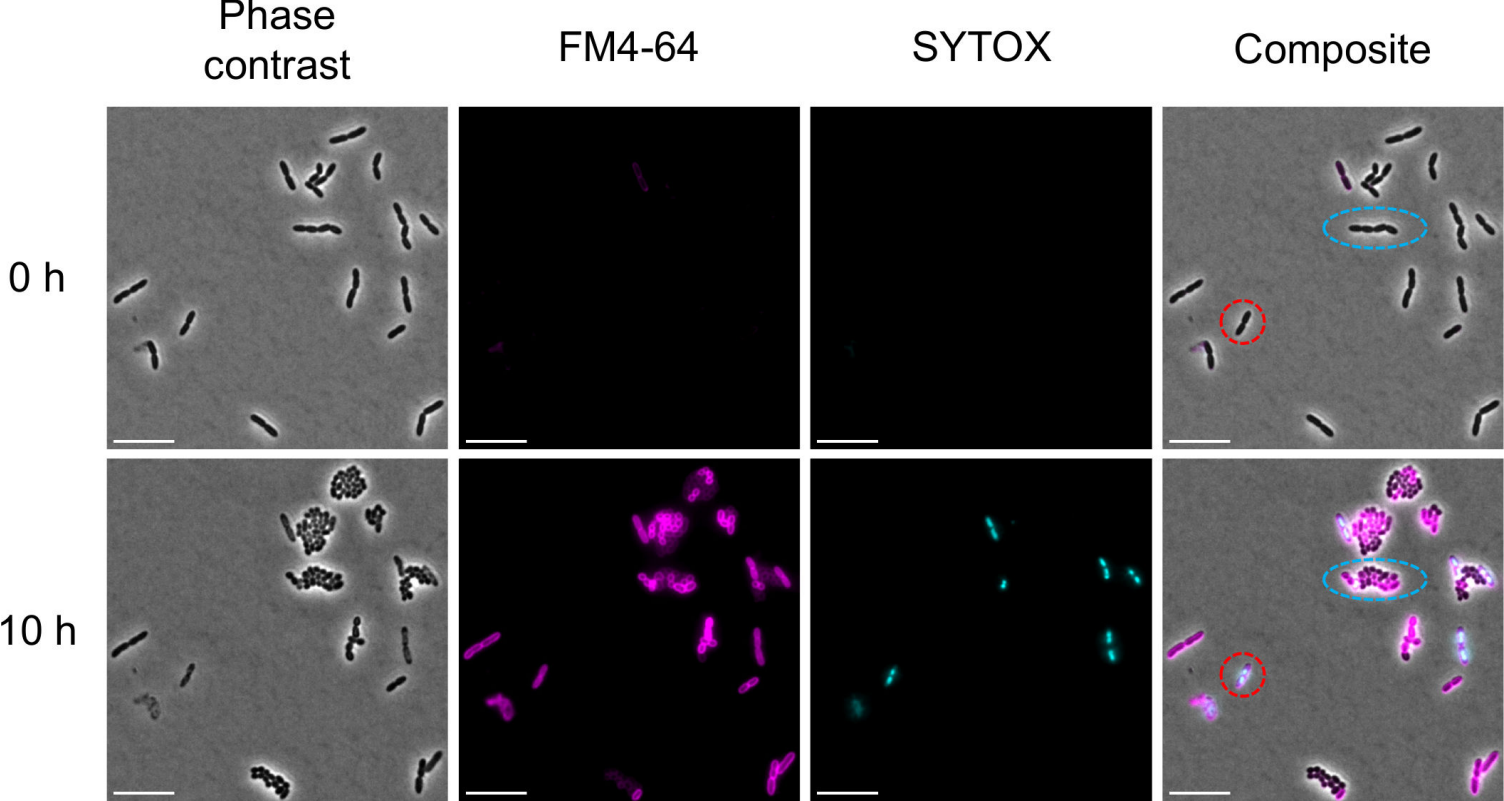
Fluorescence microscopy analysis of *P. aeruginosa* treated with pioamide. *P. aeruginosa* PAO1 cells were placed on an agarose pad containing 8× MIC of pioamide and the fluorescent dye FM4-64 (magenta) and SYTOX (light blue) to stain the membrane and demonstrate membrane permeability, respectively. *P. aeruginosa* PAO1 cells were incubated at 37°C, and growth was monitored by microscopy. The red circle indicates the loss of membrane integrity of cells; light blue indicates spherical cells. Scale bar 10 µm.

Furthermore, fluorescence microscopy analysis revealed distinct morphological changes in *P. aeruginosa* cells treated with pioamide. We observed that *P. aeruginosa* cells treated with pioamide lost their membrane integrity (Fig. 4). Interestingly, several cells underwent multiple divisions, transitioning from the typical rod shape to a spherical form, after which their growth was arrested (Fig. 4). The formation of spherical cells is commonly observed in *P. aeruginosa* treated with meropenem, an antibiotic that inhibits the synthesis of bacterial cell wall (22, 23). These spherical cells, induced by meropenem treatment, display defective cell walls and outer membranes as a survival strategy (24). In this condition, the target of the antibiotic becomes less accessible or even absent, allowing the cells to survive without acquiring resistance mutations. Our results suggest that *P. aeruginosa* transitions from the rod shape to spherical form in response to pioamide using a survival mechanism similar to that observed in meropenem-treated *P. aeruginosa*. These findings support the idea that pioamide activity is not significantly influenced by efflux pumps or porin-mediated permeability. While this may suggest that pioamide acts without entering the cell, it is also possible that it is taken up via specific uptake pathways, such as the self-promoted uptake mechanism previously described for polycationic antibiotics like colistin and aminoglycosides (25). Additionally, *P. aeruginosa* possesses species-specific outer membrane proteins like OprD, which facilitate carbapenem uptake (26). Other Gram-negative bacteria, such as *A. baumannii,* have homologs of OprD; however, deletion of *A. baumannii oprD* does not affect carbapenem resistance (27). Such structural differences in outer membrane proteins may underlie the selectivity of pioamide.

To explore the mechanism of resistance to pioamide in *P. aeruginosa*, we generated spontaneous pioamide-resistant mutant strains from the *P. aeruginosa* wild-type strain PAO1 by plating cells onto MHIIA plates containing four times the MIC of pioamide. As a result, spontaneous pioamide-resistant mutant strains were obtained with a frequency of 1.6 × 10^−7^–3.7 × 10^−7^. Whole-genome sequencing of the three mutants revealed mutations in *pmrB* (*pmrB* L18P, *pmrB* D47E, and *pmrB* c.450_451ins CAGATCTGGATCAGCGAA), which encodes a two-component regulatory system signal sensor kinase (Table S4). It is well established that specific mutations in *pmrB* confer resistance to polymyxin B and colistin, which target lipopolysaccharide (LPS) in Gram-negative bacteria and cause cell lysis. The resistance arises through the modification of lipid A by the addition of L-Ara4N, which reduces the affinity of lipid A for these antibiotics (28). Fluorescence microscopy analysis of *P. aeruginosa* treated with colistin revealed immediate cell lysis, with no visible cells in the microscopic field when an agarose pad was applied (data not shown). This finding suggests that *pmrB* mutations confer *P. aeruginosa* with resistance to both pioamide and colistin, although the mechanisms of action of these antibiotics are distinct. A recent study demonstrated that *pmrB* mutations confer cross-resistance between colistin and murepavadin (POL7080) in *P. aeruginosa* (29, 30). Murepavadin is an outer membrane-targeting peptide that specifically interacts with the LPS transporter protein LptD located on the cell surface and kills *P. aeruginosa* through a nonlytic mechanism. It has been suggested that modified LPS causes reduced murepavadin binding to LptD. It is more likely that pioamide resistance in *P. aeruginosa* is due to *pmrB* mutations inhibiting the interaction between pioamide and its target, as observed in the case of murepavadin, rather than PmrB itself being the target.

Although our study revealed important insights into the activity of pioamide and its resistance mechanisms, several questions remain unanswered due to the limited amount of pioamide currently available. Given that the resistance mechanism involves *pmrB*, which regulates LPS modifications in response to environmental stress, it is possible that the MIC of pioamide varies depending on the culture medium conditions (31). In a related context, it remains unclear whether the activity of pioamide is influenced by divalent cations, such as Ca^2+^ and Mg^2+^, which are known to affect the outer membrane integrity and antibiotic susceptibility of Gram-negative bacteria (32, 33). Therefore, MIC assays under different ionic conditions, such as in the presence or absence of divalent cations, or in minimal media like M9-MOPS, may provide further insight into the activity of pioamide. Further studies addressing these points will deepen our understanding of pioamide and contribute to the development of novel selective antibiotics against *P. aeruginosa*. Although pioamide exhibits limited potency, considering its peptidic nature, chemical derivatization is anticipated to enhance its activity (34). Remarkably, pioamide exhibited highly selective activity against *P. aeruginosa*, warranting further research.

To date, murepavadin is the only *P. aeruginosa*-selective antibacterial agent. In addition, the most effective, approved antibiotics active against *P. aeruginosa*, including recently reported compounds, are broad-spectrum agents, emphasizing the rarity of *P. aeruginosa*-selective antibiotics (Table S3) (9, 11, 29, 35–37). Considering the intrinsic resistance of *P. aeruginosa* to multiple antibiotics, such selective activity is both unusual and puzzling. Further research into the mechanism of action of pioamide may expand our understanding of potential targets for *P. aeruginosa*-selective therapeutics. Our study findings reinforce that *Photorhabdus* species are prolific producers of antibiotics with novel modes of action. Therefore, these bacterial species may remain a rich source for the discovery of new antibiotics effective against Gram-negative pathogens.

## SUPPLEMENTAL MATERIAL

Supplemental figures aem.01123-25-s0001.docx Figures S1 and S2.

Supplemental tables aem.01123-25-s0002.docx Tables S1 to S4.

ASM does not own the copyrights to Supplemental Material that may be linked to, or accessed through, an article. The authors have granted ASM a non-exclusive, world-wide license to publish the Supplemental Material files. Please contact the corresponding author directly for reuse.

## References

[1] Naghavi M, Vollset SE, Ikuta KS, Swetschinski LR, Gray AP, Wool EE, Robles Aguilar G, Mestrovic T, Smith G, Han C, et al.. 2024. Global burden of bacterial antimicrobial resistance 1990–2021: a systematic analysis with forecasts to 2050. Lancet 404:1199–1226. doi:10.1016/S0140-6736(24)01867-1 39299261 PMC11718157

[2] Sati H, Carrara E, Savoldi A, Hansen P, Garlasco J, Campagnaro E, Boccia S, Castillo-Polo JA, Magrini E, Garcia-Vello P, Wool E, Gigante V, Duffy E, Cassini A, Huttner B, Pardo PR, Naghavi M, Mirzayev F, Zignol M, Cameron A, Tacconelli E, WHO Bacterial Priority Pathogens List Advisory Group. 2025. The WHO Bacterial Priority Pathogens List 2024: a prioritisation study to guide research, development, and public health strategies against antimicrobial resistance. Lancet Infect Dis 25:1033–1043. doi:10.1016/S1473-3099(25)00118-5 40245910 PMC12367593

[3] WHO. 2025. WHO Bacterial Priority Pathogens List, 2024: bacterial pathogens of public health importance to guide research, development and strategies to prevent and control antimicrobial resistance. Available from: https://www.who.int/publications/i/item/9789240093461 10.1016/S1473-3099(25)00118-5 PMC12367593 40245910

[4] Centers for Disease Control and Prevention (CDC). 2019. Antibiotic Resistance Threats in the United States, 2019. Available from: https://www.cdc.gov/antimicrobial-resistance/data-research/threats/index.html

[5] Tobias NJ, Wolff H, Djahanschiri B, Grundmann F, Kronenwerth M, Shi Y-M, Simonyi S, Grün P, Shapiro-Ilan D, Pidot SJ, Stinear TP, Ebersberger I, Bode HB. 2017. Natural product diversity associated with the nematode symbionts Photorhabdus and Xenorhabdus. Nat Microbiol 2:1676–1685. doi:10.1038/s41564-017-0039-9 28993611

[6] Lewis K. 2020. The science of antibiotic discovery. Cell 181:29–45. doi:10.1016/j.cell.2020.02.056 32197064

[7] Leimer N, Wu X, Imai Y, Morrissette M, Pitt N, Favre-Godal Q, Iinishi A, Jain S, Caboni M, Leus IV, et al.. 2021. A selective antibiotic for Lyme disease. Cell 184:5405–5418. doi:10.1016/j.cell.2021.09.011 34619078 PMC8526400

[8] Imai Y, Hauk G, Quigley J, Liang LB, Son S, Ghiglieri M, Gates MF, Morrissette M, Shahsavari N, Niles S, Baldisseri D, Honrao C, Ma XY, Guo JJ, Berger JM, Lewis K. 2022. Evybactin is a DNA gyrase inhibitor that selectively kills Mycobacterium tuberculosis. Nat Chem Biol 18:1236–1244. doi:10.1038/s41589-022-01102-7 35996001 PMC9844538

[9] Imai Y, Meyer KJ, Iinishi A, Favre-Godal Q, Green R, Manuse S, Caboni M, Mori M, Niles S, Ghiglieri M, et al.. 2019. A new antibiotic selectively kills Gram-negative pathogens. Nature 576:459–464. doi:10.1038/s41586-019-1791-1 31747680 PMC7188312

[10] Kaur H, Jakob RP, Marzinek JK, Green R, Imai Y, Bolla JR, Agustoni E, Robinson CV, Bond PJ, Lewis K, Maier T, Hiller S. 2021. The antibiotic darobactin mimics a β-strand to inhibit outer membrane insertase. Nature 593:125–129. doi:10.1038/s41586-021-03455-w 33854236

[11] Miller RD, Iinishi A, Modaresi SM, Yoo BK, Curtis TD, Lariviere PJ, Liang LB, Son S, Nicolau S, Bargabos R, Morrissette M, Gates MF, Pitt N, Jakob RP, Rath P, Maier T, Malyutin AG, Kaiser JT, Niles S, Karavas B, Ghiglieri M, Bowman SEJ, Rees DC, Hiller S, Lewis K. 2022. Computational identification of a systemic antibiotic for gram-negative bacteria. Nat Microbiol 7:1661–1672. doi:10.1038/s41564-022-01227-4 36163500 PMC10155127

[12] Shahsavari N, Wang B, Imai Y, Mori M, Son S, Liang L, Böhringer N, Manuse S, Gates MF, Morrissette M, Corsetti R, Espinoza JL, Dupont CL, Laub MT, Lewis K. 2022. A silent operon of Photorhabdus luminescens encodes a prodrug mimic of GTP. mBio 13:e0070022. doi:10.1128/mbio.00700-22 35575547 PMC9239236

[13] Bargabos R, Iinishi A, Hawkins B, Privalsky T, Pitt N, Son S, Corsetti R, Gates MF, Miller RD, Lewis K. 2024. Small molecule produced by Photorhabdus interferes with ubiquinone biosynthesis in Gram-negative bacteria. mBio 15:e0116724. doi:10.1128/mbio.01167-24 39254306 PMC11481567

[14] Shi Y-M, Hirschmann M, Shi Y-N, Ahmed S, Abebew D, Tobias NJ, Grün P, Crames JJ, Pöschel L, Kuttenlochner W, Richter C, Herrmann J, Müller R, Thanwisai A, Pidot SJ, Stinear TP, Groll M, Kim Y, Bode HB. 2022. Global analysis of biosynthetic gene clusters reveals conserved and unique natural products in entomopathogenic nematode-symbiotic bacteria. Nat Chem 14:701–712. doi:10.1038/s41557-022-00923-2 35469007 PMC9177418

[15] Imai Y. 2025. Overproduction of secondary metabolites in Photorhabdus noenieputensis through rpoB mutations. J Biosci Bioeng 139:399–405. doi:10.1016/j.jbiosc.2025.02.004 40121163

[16] Blin K, Shaw S, Augustijn HE, Reitz ZL, Biermann F, Alanjary M, Fetter A, Terlouw BR, Metcalf WW, Helfrich EJN, van Wezel GP, Medema MH, Weber T. 2023. antiSMASH 7.0: new and improved predictions for detection, regulation, chemical structures and visualisation. Nucleic Acids Res 51:W46–W50. doi:10.1093/nar/gkad344 37140036 PMC10320115

[17] Bode HB, Reimer D, Fuchs SW, Kirchner F, Dauth C, Kegler C, Lorenzen W, Brachmann AO, Grün P. 2012. Determination of the absolute configuration of peptide natural products by using stable isotope labeling and mass spectrometry. Chem Eur J 18:2342–2348. doi:10.1002/chem.201103479 22266804

[18] Nollmann FI, Dauth C, Mulley G, Kegler C, Kaiser M, Waterfield NR, Bode HB. 2015. Insect-specific production of new GameXPeptides in Photorhabdus luminescens TTO1, widespread natural products in entomopathogenic bacteria. ChemBioChem 16:205–208. doi:10.1002/cbic.201402603 25425189

[19] Zhao L, Awori RM, Kaiser M, Groß J, Opatz T, Bode HB. 2019. Structure, biosynthesis, and bioactivity of photoditritide from Photorhabdus temperata Meg1. J Nat Prod 82:3499–3503. doi:10.1021/acs.jnatprod.9b00932 31799840

[20] Wolloscheck D, Krishnamoorthy G, Nguyen J, Zgurskaya HI. 2018. Kinetic control of quorum sensing in Pseudomonas aeruginosa by multidrug efflux pumps. ACS Infect Dis 4:185–195. doi:10.1021/acsinfecdis.7b00160 29115136 PMC5807214

[21] Cooper CJ, Krishnamoorthy G, Wolloscheck D, Walker JK, Rybenkov VV, Parks JM, Zgurskaya HI. 2018. Molecular properties that define the activities of antibiotics in Escherichia coli and Pseudomonas aeruginosa. ACS Infect Dis 4:1223–1234. doi:10.1021/acsinfecdis.8b00036 29756762 PMC6449051

[22] Trautmann M, Heinemann M, Zick R, Möricke A, Seidelmann M, Berger D. 1998. Antibacterial activity of meropenem against Pseudomonas aeruginosa, including antibiotic-induced morphological changes and endotoxin-liberating effects. Eur J Clin Microbiol Infect Dis 17:754–760. doi:10.1007/s100960050180 9923514

[23] Horii T, Kobayashi M, Sato K, Ichiyama S, Ohta M. 1998. An in-vitro study of carbapenem-induced morphological changes and endotoxin release in clinical isolates of gram-negative bacilli. J Antimicrob Chemother 41:435–442. doi:10.1093/jac/41.4.435 9598774

[24] Monahan LG, Turnbull L, Osvath SR, Birch D, Charles IG, Whitchurch CB. 2014. Rapid conversion of Pseudomonas aeruginosa to a spherical cell morphotype facilitates tolerance to carbapenems and penicillins but increases susceptibility to antimicrobial peptides. Antimicrob Agents Chemother 58:1956–1962. doi:10.1128/AAC.01901-13 24419348 PMC4023726

[25] Hancock RE, Wong PG. 1984. Compounds which increase the permeability of the Pseudomonas aeruginosa outer membrane. Antimicrob Agents Chemother 26:48–52. doi:10.1128/AAC.26.1.48 6433788 PMC179915

[26] Li H, Luo YF, Williams BJ, Blackwell TS, Xie CM. 2012. Structure and function of OprD protein in Pseudomonas aeruginosa: from antibiotic resistance to novel therapies. Int J Med Microbiol 302:63–68. doi:10.1016/j.ijmm.2011.10.001 22226846 PMC3831278

[27] Catel-Ferreira M, Nehmé R, Molle V, Aranda J, Bouffartigues E, Chevalier S, Bou G, Jouenne T, Dé E. 2012. Deciphering the function of the outer membrane protein OprD homologue of Acinetobacter baumannii. Antimicrob Agents Chemother 56:3826–3832. doi:10.1128/AAC.06022-11 22564848 PMC3393417

[28] Moskowitz SM, Ernst RK, Miller SI. 2004. PmrAB, a two-component regulatory system of Pseudomonas aeruginosa that modulates resistance to cationic antimicrobial peptides and addition of aminoarabinose to lipid A. J Bacteriol 186:575–579. doi:10.1128/JB.186.2.575-579.2004 14702327 PMC305751

[29] Srinivas N, Jetter P, Ueberbacher BJ, Werneburg M, Zerbe K, Steinmann J, Van der Meijden B, Bernardini F, Lederer A, Dias RLA, Misson PE, Henze H, Zumbrunn J, Gombert FO, Obrecht D, Hunziker P, Schauer S, Ziegler U, Käch A, Eberl L, Riedel K, DeMarco SJ, Robinson JA. 2010. Peptidomimetic antibiotics target outer-membrane biogenesis in Pseudomonas aeruginosa. Science 327:1010–1013. doi:10.1126/science.1182749 20167788

[30] Romano KP, Warrier T, Poulsen BE, Nguyen PH, Loftis AR, Saebi A, Pentelute BL, Hung DT. 2019. Mutations in pmrB confer cross-resistance between the LptD inhibitor POL7080 and colistin in Pseudomonas aeruginosa. Antimicrob Agents Chemother 63:e00511-19. doi:10.1128/AAC.00511-19 31235628 PMC6709506

[31] Chen HD, Groisman EA. 2013. The biology of the PmrA/PmrB two-component system: the major regulator of lipopolysaccharide modifications. Annu Rev Microbiol 67:83–112. doi:10.1146/annurev-micro-092412-155751 23799815 PMC8381567

[32] D’amato RF, Thornsberry C, Baker CN, Kirven LA. 1975. Effect of calcium and magnesium ions on the susceptibility of Pseudomonas species to tetracycline, gentamicin polymyxin B, and carbenicillin. Antimicrob Agents Chemother 7:596–600. doi:10.1128/AAC.7.5.596 167658 PMC429188

[33] Vaara M. 1992. Agents that increase the permeability of the outer membrane. Microbiol Rev 56:395–411. doi:10.1128/mr.56.3.395-411.1992 1406489 PMC372877

[34] Zampaloni C, Mattei P, Bleicher K, Winther L, Thäte C, Bucher C, Adam J-M, Alanine A, Amrein KE, Baidin V, et al.. 2024. A novel antibiotic class targeting the lipopolysaccharide transporter. Nature 625:566–571. doi:10.1038/s41586-023-06873-0 38172634 PMC10794144

[35] Vaara Martti, Fox J, Loidl G, Siikanen O, Apajalahti J, Hansen F, Frimodt-Møller N, Nagai J, Takano M, Vaara T. 2008. Novel polymyxin derivatives carrying only three positive charges are effective antibacterial agents. Antimicrob Agents Chemother 52:3229–3236. doi:10.1128/AAC.00405-08 18591267 PMC2533495

[36] Hernandez V, Crépin T, Palencia A, Cusack S, Akama T, Baker SJ, Bu W, Feng L, Freund YR, Liu L, et al.. 2013. Discovery of a novel class of boron-based antibacterials with activity against gram-negative bacteria. Antimicrob Agents Chemother 57:1394–1403. doi:10.1128/AAC.02058-12 23295920 PMC3591879

[37] Chain C, Sheehan JP, Xu X, Ghaffari S, Godbole A, Kim H, Freundlich JS, Rabinowitz JD, Gitai Z. 2024. A folate inhibitor exploits metabolic differences in Pseudomonas aeruginosa for narrow-spectrum targeting. Nat Microbiol 9:1207–1219. doi:10.1038/s41564-024-01665-2 38594311 PMC11087268

