# Supplementary material for: A novel homoarginine-containing cyclic peptide pioamide with selective antipseudomonal activity isolated from the nematode symbiont Photorhabdus khanii
Source: Appl Environ Microbiol. 2025 Sep 24;91(10):e01123-25. doi: 10.1128/aem.01123-25 (PMC12542766; doi:10.1128/aem.01123-25)
Supplement: Supplemental figures — Figures S1 and S2. [file aem.01123-25-s0001.docx]

**Supplementary Fig. 1| HPLC and MS analysis of pioamide.**

**a**, HPLC chromatogram of pioamide, monitored by UV absorbance at 280 nm. **b**, Mass spectrum of pioamide.

**
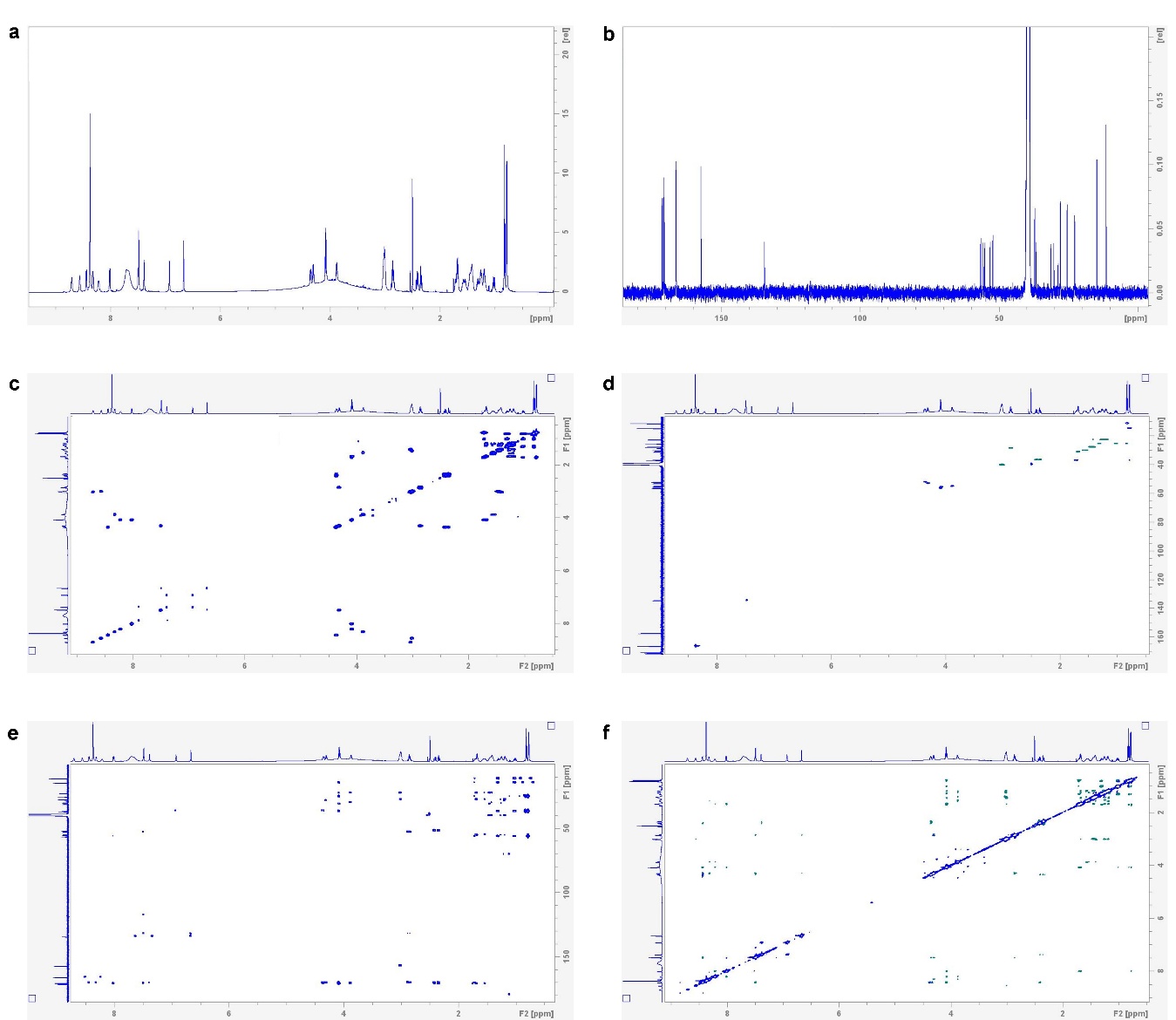
**

**Supplementary Fig. 2| NMR spectra of pioamide in DMSO-*d*_6_.**

**a**, ^1^H NMR spectrum; **b**, ^13^C NMR spectrum; **c**, ^1^H-^1^H COSY spectrum; **d**, ^1^H-^13^C HSQC spectrum; **e**, ^1^H-^13^C HMBC spectrum; **f**, ^1^H-^1^H ROESY spectrum.
