# Supplementary material for: A novel homoarginine-containing cyclic peptide pioamide with selective antipseudomonal activity isolated from the nematode symbiont Photorhabdus khanii
Source: Appl Environ Microbiol. 2025 Sep 24;91(10):e01123-25. doi: 10.1128/aem.01123-25 (PMC12542766; doi:10.1128/aem.01123-25)
Supplement: Supplemental tables — Tables S1 to S4. [file aem.01123-25-s0002.docx]

Supplementary Table 1 ^1^H and ^13^C NMR data of pioamide in DMSO-*d*_6_

|  | Type | ^1^H (mult, *J* in Hz) | ^13^C |
| --- | --- | --- | --- |
| Har_1_ |  |  |  |
| 1 | C |  | 171.2 |
| 2 | CH | 3.88 (m) | 55.1 |
| NH-2 |  | 8.32 (s) |  |
| 3 | CH_2_ | 1.58 (m), 1.54 (m) | 30.2 |
| 4 | CH_2_ | 1.25 m | 22.7 |
| 5 | CH_2_ | 1.42 m | 27.9 |
| 6 | CH_2_ | 3.03 (m) | 40.3 |
| NH-6 |  | 8.71 (br s) |  |
| 7 | C |  | 157.3 (ovl^a^) |
| NH_2_-7 |  | 7.67 (s) |  |
| Har_2_ |  |  |  |
| 8 | C |  | 171.4 |
| 9 | CH | 4.08 (ovl^a^) | 55.7 |
| NH-9 |  | 8.22 (d, 5.93) |  |
| 10 | CH_2_ | 1.68 (m) | 31.3 |
| 11 | CH_2_ | 1.19 (m) | 22.8 |
| 12 | CH_2_ | 1.45 (m) | 27.8 |
| 13 | CH_2_ | 3.01 (m) | 40.4 |
| NH-13 |  | 8.56 (br s) |  |
| 14 | C |  | 157.3 (ovl^a^) |
| NH_2_-14 |  | 7.7 (s) |  |
| Ile |  |  |  |
| 15 | C |  | 170.6 |
| 16 | CH | 4.08 (ovl^a^) | 56.5 |
| NH-16 |  | 8.02 (d, 8.1) |  |
| 17 | CH | 1.72 m | 37.1 |
| 18 | CH_2_ | 1.31 (m), 1.02 (m) | 25.5 |
| 19 | CH_3_ | 0.82 (t, 7.4) | 11.4 |
| 20 | CH_3_ | 0.78 (d, 6.8) | 14.8 |
| Asn |  |  |  |
| 21 | C |  | 171.1 |
| 22 | CH | 4.36 (m) | 52.2 |
| NH-22 |  | 8.44 (d, 7.22) |  |
| 23 | CH_2_ | 2.41 (dd, 9.6, 15.9), 2.34 (dd, 4.9, 15.1) | 36.7 |
| 24 | C |  | 170.7 |
| NH-24 |  | 7.39 (s), 6.92 (s) |  |
| His |  |  |  |
| 25 | C |  | 170.5 |
| 26 | CH | 4.31 (q, 6.84) | 53.1 |
| NH-26 |  | 7.49 (ovl^a^) |  |
| 27 | CH_2_ | 2.86 (m) | 28.6 |
| 28 | C |  | 132.2 |
| 29 | CH | 6.67 (s) | 117.8^b^ |
| 30 | CH | 7.49 (ovl^a^) | 134.5 |

^a^Overlapped signals.

^b^Assigned by the HSQC correlation.

Supplementary Table 2 MIC and cytotoxicity of pioamide

| Strain | µg/ml |
| --- | --- |
| Pathogenic bacteria (MIC) |  |
| *Pseudomonas aeruginosa* PAO1 | 128 |
| *Pseudomonas aeruginosa* P*Δ*6-Pore | 128 |
| *Pseudomonas aeruginosa* P*Δ*6-Pore +100 µM IPTG | 128 |
| *Escherichia coli* MG1655 | >1,024 |
| *Klebsiella pneumoniae* ATCC 700603 | >1,024 |
| *Acinetobacter baumannii* ATCC 17978 | >1,024 |
| *Staphylococcus aureus* HG003 | >1,024 |
| Human cell line (IC_50_) |  |
| HepG2 | >1,024 |
| FaDu | >1,024 |

Supplementary Table 3 Spectrum of antibacterials active against *P. aeruginosa*

| Antibacterials | MIC | | | | | | Target molecules | Reference | |
| --- | --- | --- | --- | --- | --- | --- | --- | --- | --- |
|  | Gram-negative | | | |  | Gram-positive |  |  |  |
|  | *P. aeruginosa* | *E. coli* | *K. pneumoniae* | *A. baumannii* |  | *S. aureus* |  |  |  |
| Murepavadin | 0.004  (PAO1) | >64  (ATCC 25922) | >64  (ATCC 13883) | >64  (DSM3008) |  | >64  (ATCC29213) | LptD | 29 | |
| Pioamide | 128  (PAO1) | >1,024  (MG1655) | >1,024  (ATCC 700603) | >1,024  (ATCC 17978) |  | >1,024  (HG003) | ND | This study | |
| Darobactin | 2  (PAO1) | 4  (MG1655) | 4  (ATCC 700603) | 8  (ATCC 17978) |  | >128  (HG003) | BamA | | 9 |
| Dynobactin | 8  (PAO1) | 16  (MG1655) | 64  (AR347) | 16  (ATCC 19606) |  | >1,000  (HG003)) | BamA | | 11 |
| Polymyxin B | 1  (ATCC 27853) | 1  (ATCC 25922) | 1  (CCUG45421) | 1  (ATCC 19606) |  | >32  (ATCC 25923) | LPS | | 35 |
| AN3365 | 1  (PAO1) | 1  (ATCC 25922) | 1  (1534) | 1  (ATCC 15473) |  | 4  (ATCC 29213) | LeuRS | | 36 |
| Fluorofolin | 6.25  (PAO1) | 0.625  (MG1655) | 6.25  (ATCC 438165) | 31.25  (BAA-125) |  | 0.4  (USA300) | DHFR | | 37 |

The MIC values of each antibiotic were adopted from reference studies. ND; Not determined. The numbers in parentheses refer to the strain identifiers of the bacterial strains used in the MIC assays.

Supplementary Table 4 Comparison of whole genome sequence data of *P. aeruginosa* PAO1 and pioamide-resistant mutants

| Strain | Position^a^ | Mutation | Annotation | Gene | Description |
| --- | --- | --- | --- | --- | --- |
| PAO1 | 1,467,482 | +G | intergenic (‑162/+6) | *PA1352* ← / ← *PA1353* | hypothetical protein/hypothetical protein |
|  | 1,467,484 | +G | intergenic (‑164/+4) | *PA1352* ← / ← *PA1353* | hypothetical protein/hypothetical protein |
|  | 3,603,486 | G→C | V54L (GTC→CTC) | *PA3214* → | hypothetical protein |
| Mutant-1 | 1,467,482 | +G | intergenic (‑162/+6) | *PA1352* ← / ← *PA1353* | hypothetical protein/hypothetical protein |
|  | 1,467,484 | +G | intergenic (‑164/+4) | *PA1352* ← / ← *PA1353* | hypothetical protein/hypothetical protein |
|  | 5,364,812 | T→C | L18P (CTG→CCG) | *pmrB* → | two‑component regulator system signal sensor kinase PmrB |
| Mutant-2 | 1,467,482 | +G | intergenic (‑162/+6) | *PA1352* ← / ← *PA1353* | hypothetical protein/hypothetical protein |
|  | 5,365,209 | (CAGATCTGGATCAGCGAA)_1→2_ | coding (450/1434 nt) | *pmrB* → | two‑component regulator system signal sensor kinase PmrB |
| Mutant-3 | 3,603,486 | G→C | V54L (GTC→CTC) | *PA3214* → | hypothetical protein |
|  | 5,364,900 | C→A | D47E (GAC→GAA) | *pmrB* → | two‑component regulator system signal sensor kinase PmrB |

^a^Mutation analysis was conducted by using reference sequence of *P. aeruginosa* PAO1 genome data deposited in NCBI. Mutations not common among 4 strains are displayed.
